# Clinical Characteristics and Outcomes in Patients Aged ≥80 Years Treated in the Intensive Care Units of a Large Multispecialty Metropolitan Hospital in Poland

**DOI:** 10.3390/jcm15010306

**Published:** 2025-12-31

**Authors:** Wojciech Bogdański, Martyna Szeląg, Miłosz Jankowski, Konstanty Szułdrzyński

**Affiliations:** Department of Anaesthesiology and Intensive Care, National Medical Institute of the Ministry of the Interior and Administration, 137 Wołoska Street, 02-507 Warsaw, Poland; martyna.szelag@pimmswia.gov.pl (M.S.); milosz.jankowski@pimmswia.gov.pl (M.J.); konstanty.szuldrzynski@pimmswia.gov.pl (K.S.)

**Keywords:** intensive care, aging, frailty

## Abstract

**Backgrounds/Objectives**: The aging of the population is reflected in the increasing number of elderly patients admitted to intensive care units (ICUs), where assessing prognosis and the potential benefit of intensive care is challenging. The aim of this study was to clinically characterize ICU patients aged ≥80 years in the National Medical Institute of the Ministry of Internal Affairs and Administration in Warsaw, Poland. **Methods:** We retrospectively analyzed ICU patients admitted between 2018 and 2022, considering comorbidities, prognostic scores, the treatment methods and outcomes. **Results:** We included 476 patients (median age 84 [range 80–103 years], female 54.4%, median ICU stay 8 days) with a high incidence of various comorbidities. The overall risk of death was very high (76.4%) but was independent of sex and, surprisingly, of age. Advanced frailty was common, as indicated by the Clinical Frailty Scale (CFS) score (median 7, *n* = 189), which was identified as a significant risk factor for death independent of age, sex, and APACHE score (odds ratio for the 1-point CFS increase: 1.08, 95% CI 1.01–1.15, *n* = 103), but not of SAPS and SOFA scores. Organ support techniques were frequently used (invasive mechanical ventilation in 90.9%, pharmacological cardiovascular support in 83.2%, and renal replacement therapy in 14.1% of patients), with high associated mortality rates (80%, 79%, and 88%, respectively). **Conclusions**: Our results confirm the value of the prognostic scales used on admission to the ICU, but also highlight the need for individualized assessment of the expected benefit of ICU treatment in elderly patients, considering specific comorbidities, previous treatment and frailty.

## 1. Introduction

Life expectancy has been increasing in recent decades, among both men and women, and the rate of population aging is much faster than in the past. It is estimated that by 2050, the number of people over 65 will increase and make up 16% of the world’s population, with a higher percentage in developed societies [1]. The growing problem of aging populations has led the WHO to declare the current decade the Decade of Healthy Aging [2].The group of people over 65 is also growing in the Polish population; in 2022 it increased by 178,000 people, reaching 7.3 million, or 19.5% of the total population (in 1990 the elderly accounted for 1/10 of the population). The growth in the number of aged people (80 and older) has also accelerated. While in 2000, the group of people reaching this age numbered 774,000 (2% of the total population), in 2022 it reached more than 1.6 million, accounting for 4.2% of Poland’s total population [3]. According to the Central Statistical Office’s 2023–2060 forecast, Poland’s population is expected to decline steadily, with a large increase in the number of people aged 85 and over (compared to 2022, this number will increase by at least 83%) [4].

Increasing life expectancy is, unfortunately, often associated with multimorbidity, the need for frequent hospitalizations and the use of invasive treatments in older patients. The problem of caring for patients over 80 particularly affects intensive care units (ICUs), where people in this age group account for 5% to as much as 20% of patients in Europe [5,6,7].

The objective of this study was to clinically characterize a cohort of patients aged 80 years or older hospitalized in the ICUs of a large multispecialty hospital in Warsaw, Poland, between 2018 and 2022.By examining the therapeutic interventions utilized and the resulting clinical outcomes, this research aims to provide a comprehensive analysis with a specific focus on mortality and its clinical determinants(predictors).Despite being studied before in other populations, this evidence from many sources is important for guiding individualized ICU admission and treatment decisions in the very elderly.

## 2. Materials and Methods

The study retrospectively analyzed the medical records of patients aged ≥80 years hospitalized in the Department of Anesthesiology and Intensive Care (3 ICUs totaling 30 beds) of the National Medical Institute (formerly Central Clinical Hospital) of the Ministry of the Interior and Administration (PIM MSWiA; total number of beds ~800) in Warsaw, Poland, between 1 January 2018 and 31 December 2022. Patients hospitalized for surgical indications were included, as well as others, excluding cardiac surgery patients, who were treated in a separate ICU. Decisions regarding ICU admission were made by specialists in anesthesiology and intensive care. Information was collected on demographics; priority for admission according to the guidelines of the Polish Society of Anesthesiology and Intensive Care (PTAiIT), reflecting the possible benefit for the patient of admission to the ICU (1—very high, 2—high, 3—difficult to predict, 4—very low); place of previous treatment (medical [non-surgical] or surgical ward or hospital emergency department [ED] of the same or another hospital); comorbidities; and scores on scales used to estimate patients’ prognosis on ICU admission: APACHE (Acute Physiology and Chronic Health Evaluation II), SAPS II (Simplified Acute Physiology Score II) and SOFA (Sequential Organ Failure Assessment) [8,9]. Diagnoses of comorbidities made or assumed based on interviews and/or medical records by attending physicians were accepted, while obesity was determined on the basis of body mass index (BMI) ≥ 30 kg/m^2^. Retrospective assessment was attempted with the Clinical Frailty Scale (CSF) based on available data [10]. Treatment in the ICU was analyzed focusing on organ support and determining whether mechanical lung ventilation, renal replacement therapy and pharmacological support of cardiovascular function were provided. The risk of death during ICU stay and the duration of ICU hospitalization were assessed. Information was also collected on whether a protocol for avoiding futile therapy was implemented in accordance with the guidelines for dealing with the lack of effectiveness of sustaining organ function in patients deprived of the ability to make conscious statements of will in intensive care units and with the position of the Society of Polish Internists Working Group on Futile Therapy in Internal Medicine Departments [11,12].

Statistical analysis was carried out using Microsoft Excel, (Microsoft 365, Microsoft Corporation, Redmond, WA, USA), IBM SPSS Statistics version 29.0.2.0 (IBM Corp., Armonk, NY, USA and TIBCO and Statistica version 13.3 (TIBCO Software Inc., Palo Alto, CA, USA). Qualitative variables were presented as counts and percentages and compared using the Chi-square test, its variations or Fisher’s exact test, depending on the assumptions met. The distribution of continuous quantitative variables was evaluated with the Shapiro–Wilk test. Variables with normal distribution were compared using ANOVA and Student’s *t*-test for uncorrelated variables, otherwise Kruskal–Wallis and Mann–Whitney tests were used. We assessed predictors of death using multivariate logistic regression. Only patients with complete datasets for the variables under study were considered, and the following independent variables were included: age, sex, and data collected at ICU admission showing significant differences between survivors and non-survivors in the comparative analysis.

## 3. Results

**Figure 1:**
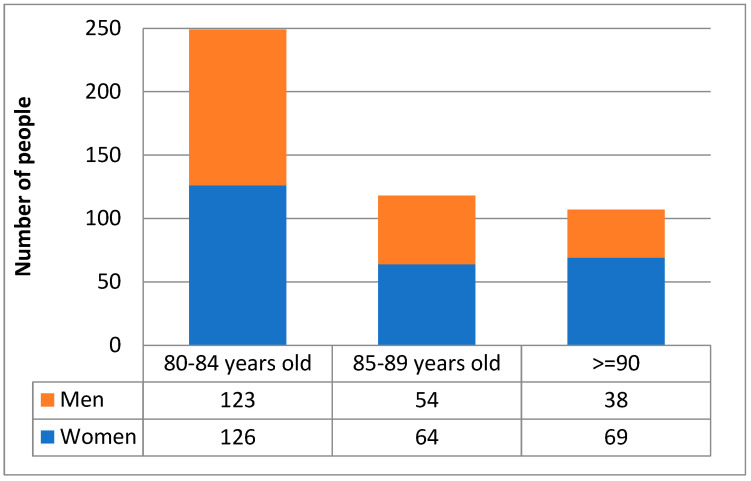
Demographic structure of the study population.

**Figure 2:**
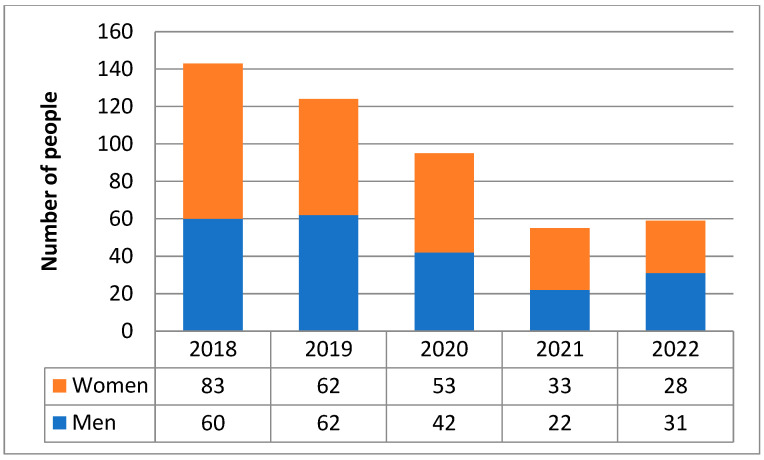
Numbers of patients aged ≥80 years admitted to the ICU in 2018–2022.

Between 2018 and 2022, 4045 patients were hospitalized in the ICUs of PIM MSWiA, of whom 476 (11.77%) were aged ≥80 years. Within this group, the largest proportion of patients was 80–84 years old (*n* = 249; 52.3%), while a minority were patients 85–89 years old (*n* = 120; 25.2%) and over 90 years old (*n* = 107;22.5%). The entire group of patients aged ≥80 years, as well as each age subgroup, was dominated by women; the female preponderance was greatest among patients over 90 (Figure 1, Table 1). Among patients aged 80+, women were significantly older than men (median [range] ages were 85 [80–103] years in women and 84 [80–98] years in men, *p* = 0.021). In the analyzed time frame, a gradual decrease in the number of elderly patients hospitalized in the ICU can be observed in successive calendar years (Figure 2).

### 3.1. ICU Admission Priorities and Previous Place of Care

**Figure 3:**
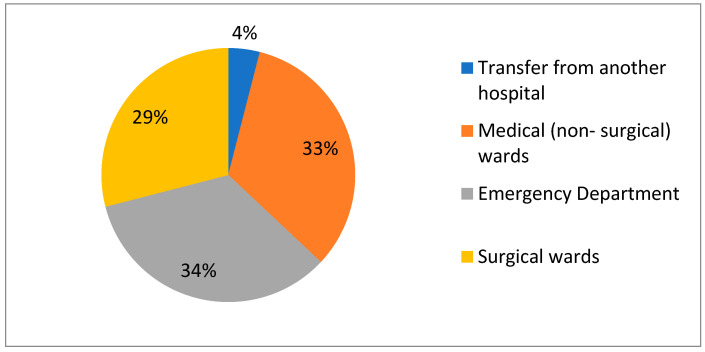
Departments referring patients aged ≥80 years to ICU in 2018–2022.

The majority of patients (*n* = 324; 68.1%) were assessed on admission to the ICU according to the priorities proposed by the PTAiIT. Among patients who had a specific admission priority, the largest number of patients received priority 3 (35%), while patients assigned priority 3 and 4 accounted for well over half of this group (60%). There were no significant differences regarding admission priority among age subgroups or between patients who died and survived (Table 1). Due to the multispecialty nature of the hospital, both patients previously treated in medical (non-surgical) and surgical wards, as well as those directly transferred from the ED, were admitted to the ICU. In the study group, 155 patients came from medical (non-surgical) wards, 140 patients came from surgical wards, and 160 patients were admitted directly from the ED, with the remaining 21 patients admitted from other hospitals (Figure 3).

**Figure 4:**
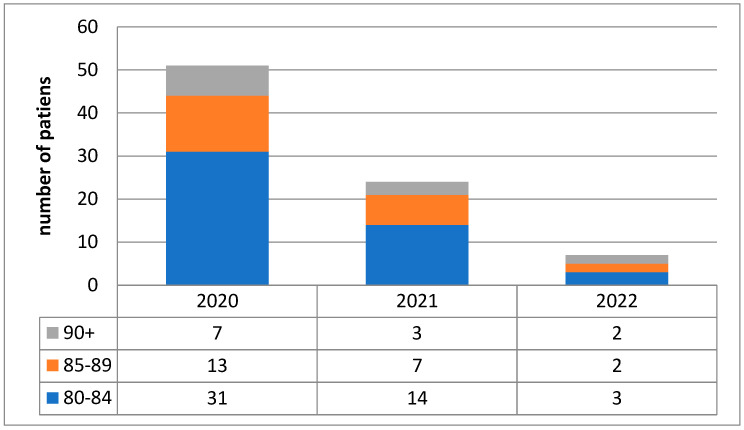
Number of COVID-19 patients admitted to the ICU between 2020 and 2022.

COVID-19 was one of the causes of hospitalization for 82 (17.2%) patients. The largest proportion of patients aged ≥80 years with confirmed SARS-CoV-2 infection was admitted to the ICU in 2020 (*n* =51; 54%); in 2021, there were 24 patients (44%), and in 2022 there were 7 (12%)(Figure 4).

### 3.2. Comorbidities and Assessment of Prognosis

**Figure 5:**
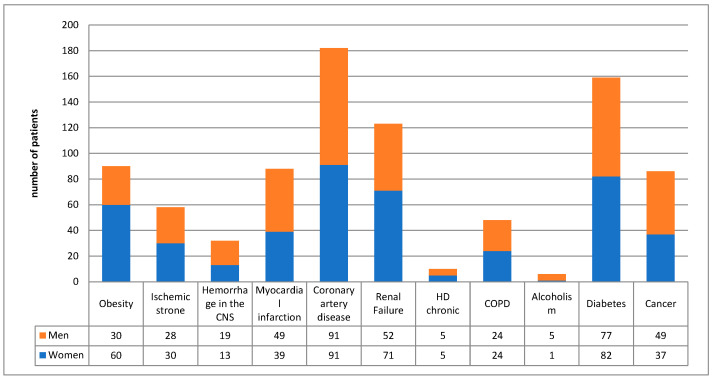
Comorbidities in patients aged ≥80 years admitted to the ICUs.

Among patients admitted to the ICU, multiple internal medicine burdens were observed (Figure 5), the most common of which were ischemic heart disease (*n* = 182; 38.2%) and diabetes (*n* = 159; 33.4%). A total of 25.8% of patients suffered from renal failure (*n* = 123), while 10 (2.1%) patients had chronic hemodialysis (prior to ICU admission). Cancer (*n* = 89; 18.7%) and chronic obstructive pulmonary disease (*n* = 48; 10.1%) were also common comorbidities. Obesity was present in 90 (32.1%) of the 280 patients with available data to calculate body mass index. In 259 (54.41%) patients, two or more of the above diagnoses were made. Only 97 (20%) patients were not burdened with any of the above diseases during ICU admission.

Median scores on the scales used to assess prognosis on ICU admission were 27 points on the APACHE scale (data calculated for *n* = 244), 67 points on the SAPS scale (*n* = 219) and 12 points on the SOFA scale (*n* = 231), corresponding to 35–55%, 75% and 85–95% mortality risk, respectively. The frailty scale score (CFS) was assessed on the basis of available medical history data, yielding scores in 189 (40%) subjects; the median was 7 (range 2 to 9). Detailed data are shown in Table 1.

### 3.3. Organ Support in the ICU

**Figure 6:**
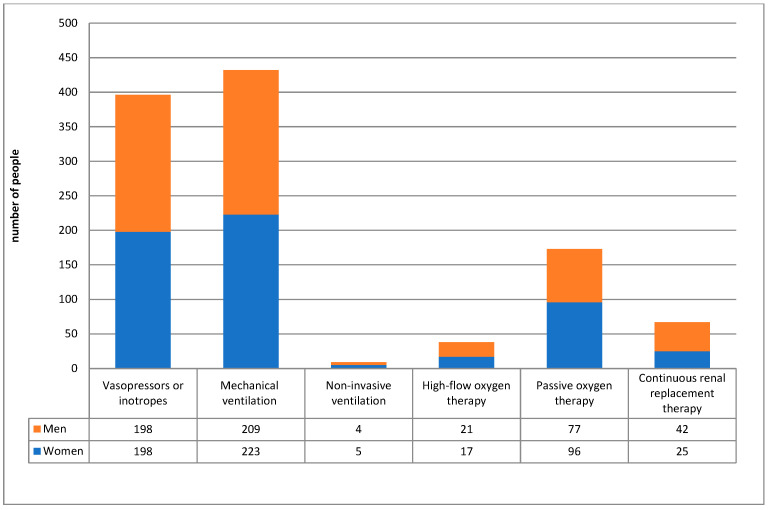
Treatment used in the ICU.

In the analyzed group of patients, intensive treatment methods were used in the form of conventional (invasive) mechanical ventilation (*n* = 432; 90.8%), non-invasive ventilation (*n* = 9; 1.9%), high-flow oxygen therapy (*n* = 38; 8.0%), cardiovascular support with infusion of catecholamines (*n* = 396; 83.2%), and renal replacement therapy (*n* = 67;14.1%) by continuous veno-venous hemodialysis or hemodiafiltration. Detailed data are shown in Table 1 and Figure 6.

### 3.4. Risk of Death in the ICU

The mortality rate in patients aged ≥80 years was 76.4%, which was significantly higher compared to younger patients hospitalized in the ICU during the period analyzed, of whom 37.1% died (*p* < 0.001). Among patients aged 80–84, the risk of death was 72%; in the group aged 85–90, it was 86%, and over 90–77%, while among all patients (regardless of age) treated in the ICU during the analyzed period, 49% died. The risk of death did not differ between women and men aged ≥80 years (74.0% vs. 79.3%, *p* = 0.181). In individual calendar years from 2018 to 2022, mortality in this age group was 89.5%, 77.4%, 58.9%, 90.9% and 55.9%, respectively, while among all hospitalized patients, it was 49%, 39%, 39%, 50% and 35%, respectively.

There was no difference in the risk of death between patients over 80 admitted from surgical departments (76.4%) and other departments (77.1%; *p* = 0.876). Among post-operative deaths (*n* = 116), significantly more deaths were related to emergency surgery (77.6%). In patients admitted to the ICU from the ED, the risk of death was 71.7% and was not significantly different compared to other patients (79.0%, *p* = 0.074). Among COVID-19 patients, the mortality rate for patients over 80 years of age reached 71% and 88% in 2020 and 2021, respectively, while in 2022 it was 71%. Mortality among all COVID-19 patients did not differ significantly from that in the overall non-COVID group (75.6% versus 76.6%, *p* = 0.949). During the pandemic (from March 2020 onwards), the death rate in non-COVID patients was significantly lower than in the pre-pandemic period (62.6% [67/207] versus 81.8% [234/286]; *p* < 0.001).

Taking into account chronic comorbidities, 85% of those with chronic obstructive disease, 77% of those with of those with chronic kidney disease (90.0% of patients on chronic dialysis), 76% with diabetes and 74% of patients with ischemic heart disease died.

Considering the organ support techniques used, among those over 80 years of age, 80% of patients undergoing invasive mechanical ventilation, 79% of patients receiving vasopressors and/or inotropic drugs and 88% of patients receiving renal replacement therapy died.

A comparison of patients aged ≥80 years who died during ICU hospitalization with survivors in this age group is shown in Table 1. No significant differences were observed with regard to age, gender, comorbidities and duration of ICU hospitalization. Patients who eventually died during their ICU stay had significantly higher scores on the APACHE, SAPS, SOFA and CSF scales at ICU admission compared to survivors. In the group of non-survivors, invasive mechanical ventilation, vasoconstrictor and/or inotropic drugs and renal replacement therapy were used more often, while non-invasive respiratory support techniques were used less often. Logistic regression analysis in patients with available data indicated that frailty (CFS) was a risk factor for death, independent of age, gender and APACHE scale scores, but not SAPS and SOFA scales. Scores on the APACHE, SAPS and SOFA scales were risk factors for death independent of age, sex and CFS. Age and gender had no statistically significant effect on the risk of death (Table 2).

### 3.5. Duration of Treatment in the ICU and the Issue of Futile Therapy

The median length of ICU hospitalization was 8 days (7 days in patients who died in the ICU and 8 days in patients who survived). The majority (72%) of patients aged ≥80 years admitted to the ICU stayed in the unit for up to 14 days, and half of them for up to 7 days. In a much smaller percentage of patients, the duration was 15–28 days (16%) or more than 28 days (11%). In all these subgroups, mortality remained similar (74–76%). A protocol to limit futile therapy was established for only 66 patients (14%).

## 4. Discussion

This study analyzed the mortality of patients aged ≥80 years in the Intensive Care Units of a large multispecialty metropolitan hospital in Poland. Factors affecting the patient before hospitalization in the ICU (qualifying unit, COVID-19, comorbidities, CFS) were taken into account, and the therapeutic methods used and the results of treatment were considered.

### 4.1. Share of Patients Aged 80 Years and Older in the ICU Patient Population and the Risk of Death

In many European countries and in the US, as well as in Australia and New Zealand, the population is gradually aging. This is reflected in the gradual increase in the percentage of admissions to hospitals and ICUs of patients > 80 years of age, which is currently 8.9–19.2% depending on the region, and within this range is the 12.3% percentage of patients ≥ 80 years of age hospitalized at our center [13,14]. In some studies conducted in the elderly, there were clear differences between the number of male and female patients in intensive care units (53% and 48%, respectively) and gender-dependent differences in the age of patients (men were younger than women) [15]. In the population we analyzed, the female preponderance increased with age (Figure 1), and women were significantly older, although median ages differed by only one year. In an international review of studies involving patients over 90 years of age, the median hospital mortality rate was 25.55% [16]. In our analysis, the death rate was higher than in the mentioned study, at 76%, but was within the range reported by a systematic review including 36 studies, which found that the mortality rate of elderly COVID-19 patients ranged from 8 to 90% depending on the center [17]. In our analysis, there was no significant difference in risk of death by gender.

### 4.2. Assessing the Prognosis of Elderly Patients Admitted to the ICU

In considering the prognosis of ICU patients, concomitant diseases including chronic renal failure, chronic heart failure, diabetes, chronic obstructive pulmonary disease and dementia syndromes are undoubtedly an important factor. Studies show that multimorbidity, defined as the comorbidity of two or more diseases, affects up to 90% of patients aged >85 years [18]. A multicenter study of elderly COVID-19 patients treated in the ICU confirmed a significant increase in 90-day mortality in the group of patients with diabetes (64%, compared to 56% in the group of patients without diabetes) [19]. This is likely related to the development of cardiovascular complications (e.g., hypertension, heart failure) and kidney damage. Patients with diabetes also repeatedly received a higher CFS score. As for chronic heart failure, studies suggest that although it increases the mortality of ICU patients on its own, compared to other factors (e.g., age, SOFA, other comorbidities), it is not as strong a predictor [20]. Also, chronic kidney disease, both requiring renal replacement therapy and in less advanced stages, significantly increased the risk of death [21]. In our analysis, comorbidities considered individually were not significantly more prevalent in those who eventually died, while the cited studies also included people under the age of 80.

Standard scales for assessing the risk of death in the ICU may also be applicable to elderly patients. In the group of patients we described, scores on the APACHE II, SAPS and SOFA scales at ICU admission were significantly higher in patients who died compared to survivors. This is consistent with the results of a study conducted among patients >65 years of age, in which predictions based on the APACHE and SOFA scales did not differ significantly from actual mortality, as well as a study confirming the utility of the SAPS II scale in the elderly [22]. In the analysis we presented, the actual risk of death was higher than that corresponding to the median score on the APACHE scale, while it was lower than that predicted based on the median on the SAPS and SOFA scales. The value of the aforementioned scales in predicting ICU survival among populations ≥80 years of age therefore appears limited and this indicates the need to consider other prognostic factors.

Predicting the course of disease and the prognosis of elderly patients over the age of 80 often poses considerable difficulties, which, combined with the truncated number of ICU beds, as well as ethical and economic issues, encourages the ongoing search for prognostic factors relevant to this age group that are objective and based on high-level scientific evidence, as well as criteria for deciding on treatment methods. A tool that has proven effective for patients over 65 during the COVID 19 pandemic, among others, and whose effectiveness has been proven in numerous VIP2 papers, is the frailty scale (CFS) [23]. Unfortunately, there is no consistent approach as to how a high CFS score unequivocally predicts a poor prognosis. The NICE guideline authors proposed a UK cutoff of 5 points on the CSF [24]. However, this value applies to the screening of COVID-19 patients, and does not exclude the use of intensive care approaches in patients with a higher score who may benefit from the treatment. In contrast, a multicenter cohort study conducted in 2020 in Australia and New Zealand analyzing 5607 patients with pneumonia challenges the above guidelines and argues that only the highest CSF scores, 7 and 8 points, are associated with higher mortality [25]. In contrast, the results of VIP 1 and VIP 2 show that the association between CSF and patients > 80 years of age is non-linear, with significant increases in mortality occurring in patients who have already scored 6 [26].

The CFS was selected for use in this study as it is the most widely applied scale in ICU patients, while other tools are employed in different populations (e.g., patients with heart failure [27]) and settings [28]. Furthermore, CFS is based on clinical judgement, as opposed to self-reporting or measurements of cognitive and physical performance, as these cannot be carried out retrospectively or on ICU patients. Among the patients analyzed in our study, CSF was assessed retrospectively based on medical history; the median CFS in those with available data was 7 points and scores were significantly higher in patients who died compared to survivors (medians of 7 and 6 points, respectively, *p* = 0.008). In addition, logistic regression analysis suggested that CFS was a predictor of death independent of age, gender and APACHE scale scores, but not SAPS and SOFA scales. However, we did not have complete data due to the failure to conduct frailty assessments at our center prior to the COVID-19 pandemic, as well as the frequent difficulty of medical staff and patients’ families in reliably determining a patient’s fitness prior to admission. There is no doubt, however, that the CFS is now a useful tool, the use of which assesses the risk of death and helps make therapeutic decisions, especially in correlation with other factors, such as scores on other scales for assessing prognosis and taking into account concomitant diseases [29].

### 4.3. Treatment Methods Used in the Elderly ICU Patients

The aging of the population and the associated transformation of acute diseases into chronic ones, coupled with the wider availability of new treatments, force the constant need to find new solutions in healthcare systems, raising questions about the limits of therapy for elderly patients. Published data indicate that patients > 90 years of age are more likely to not undergo advanced, invasive treatments, i.e., renal replacement therapy, invasive mechanical ventilation and the use of vasoactive drugs [30]. Also, in the group of patients > 80 years of age, there is a more frequent reduction in the implementation of invasive mechanical ventilation than in younger patients (28.3% vs. 37.8%) [31]. The need for mechanical ventilation in patients aged 85+ is associated with both a high in-hospital mortality, reaching 64.1% according to available studies, and frequent need for prolonged mechanical ventilation in patients discharged from the ICU (15.1%) [32]. For continuous renal replacement therapy, the available literature remains inconclusive. Patients who have experienced an episode of acute kidney injury have a higher risk of developing chronic kidney disease. A meta-analysis published in 2008 found that 31.3% of elderly patients (≥65 years old) who survived did not have their kidney function returned to normal compared to 26% of younger patients [33]. In one retrospective study involving a group of elderly patients (≥65 years) undergoing CRRT, improvement in renal function was observed in only 6% and permanent loss of renal function in 23% of patients [34]. In addition, both short-term (during hospital stay or within 1 week of discharge) and long-term mortality rates among patients undergoing CRRT were higher among patients older than 85 than in younger patients (70% vs. 55% and 50% vs. 33%, respectively) [35]. However, there are also reports that there are no significant differences in dialysis between older and younger patients at discharge from the intensive care unit (31.9% vs. 35.8%) and from the hospital (18.5% vs. 24.2%) [36]. Our study confirms the very high risk of death among patients aged ≥80 years requiring mechanical ventilation (79.8%) and/or CRRT (88%). Due to the unavailability of data, it was not possible to analyze the incidence of permanent deterioration or loss of renal function and the need for dialysis or mechanical ventilation after ICU discharge.

### 4.4. Futility Assesment

The remarkably high ICU mortality rate documented in our cohort (76.4%) underscores the imperative for a nuanced discourse on medical futility in elderly patients. The findings of this study indicate that the high mortality burden is not merely a function of chronological age, but rather a reflection of severe clinical sickness, as evidenced by high APACHE II, SOFA, and SAPS II scores, alongside a significant burden of comorbidities and physiological reserve reflected by frailty (CFS).

Notably, none of the patients in our study had pre-existing Do Not Resuscitate (DNR) orders or advance directives in place at the time of ICU admission. In the absence of such directives, our clinical decision-making regarding the withholding or withdrawal of life-sustaining treatment followed national Polish guidelines on avoiding medical futility. However, these guidelines currently lack granular clinical criteria, leaving the determination of futility to the expert judgment of specialists in anesthesiology and intensive therapy [11]. Moreover, there is an absence of Polish guidelines dedicated to the management of elderly patients in the ICU.

Patients aged ≥80 years are often disqualified from ICU treatment by an intensive care specialist, even if they have been prequalified by a medical (non-surgical) unit physician. According to one observational study, up to 73.3% of patients are determined to be “too healthy” (28%) or “too sick” (44%) for ICU admission [37]. However, the dilemma regarding the management of patients ≥ 80 years of age does not end with this decision. Studies have attempted to answer the question of how long ICU hospitalization allows assessment of the effectiveness of the therapy used, which should precede the decision to further intensify or limit and withdraw advanced treatments, i.e., renal replacement therapy, parenteral nutrition or mechanical ventilation. The VIP 2 study set this limit at 7 days but stressed that this is variable on an individual basis and should be considered on a case-by-case basis [38,39]. It is also important to ask what the future holds for patients over 80 years of age being discharged from intensive care units. According to the available literature, 6-month survival in this group was only 30–40% [40]. Another important issue is the quality of life of these patients, which, compared to the pre-hospitalization period after discharge from the hospital, is often significantly worse due to impaired performance, loss of autonomy and independence, and depressive disorders developing against this background. However, this is a very individual issue that should only be considered as one component of the decision-making process. In 2025, the ESICM guidelines for the management of very old patients in intensive care units were published, which emphasize that age alone should not be the basis for deciding to limit life-sustaining therapy. This is consistent with the earlier national guidelines [24,41]. Whenever possible, the patient’s previously expressed preferences and the expected quality of life should always be taken into account. In addition, further therapy and goals of care should be reassessed at regular intervals [42]

### 4.5. Limitations of the Study

The present study is subject to limitations related to its retrospective, single-center design, which may include incomplete or inaccurate data, and selection bias.

Due to the retrospective nature of the study, analysis on scales (APACHE, SAPS, SOFA, CSF) designed to assess patient prognosis during ICU stay is incomplete. CFSwas determined retrospectively based on available medical documentation containing data from interviews with patients’ relatives.This method may be less accurate than a prospective assessment performed at the time of admission. During ICU stay, we were unable to accurately assess the duration of mechanical ventilation weaning or weaning management strategies due to a lack of standardized protocols and granular data during the study period. Due to the unavailability of records of patients who were discharged from the hospital, the direction of discharge and the time of survival and quality of life after ICU treatment were also not recorded.

Moreover, because of the single-center design, our data represent only those patients who gained access to this specific ICU. Consequently, the results may be subject to selection bias related to local referral patterns and admission thresholds. Additionally, the exclusion of cardiac surgery patients, who were treated in a separate unit, may have introduced selection bias by omitting a subgroup with distinct risk profiles and potentially different outcomes. It should also be noted that for part of the period analyzed, PIM MSWiA served as a dedicated hospital for the treatment of patients infected with coronavirus. The inclusion of the pandemic period may influence our findings due to altered admission criteria, bed availability, and reorganization of care. This is reflected in the 59% declinein patient numbers from 2018 to 2022, which may limit the generalizability of our results to non-pandemic conditions. On the one hand, this increased the proportion of severe COVID-19 patients requiring treatment in the ICU setting, especially in the early months of the pandemic. However, due to high occupancy rates and the need for ECMO therapy, for which, among other things, an age criterion was used for inclusion, the number of patients over the age of 80 eligible for treatment in the ICU setting decreased in the following months. Therefore, it is possible that the pandemic burden has affected non-COVID patients aged 80 years and over. However, the ICU mortality rate for this age group was lower during the pandemic than in the pre-pandemic period. This suggests that admission criteria were more stringent rather than the pandemic having an inadvertent impact on the quality of care within the ICU.

Finally, in our study we employed a complete-case analysis for the multivariate logistic regression, which resulted in varying sample sizes across different models. This variation may have influenced the statistical power for certain variables.

## 5. Conclusions

ICU patients aged ≥80 years at our center are a heterogeneous group with various comorbidities and a very high mortality. The risk of death for these patients was independent of sex and, surprisingly, of age. Patients often had advanced frailty, as indicated by a high score on the CSF scale; the available data showed that the CSF score was a risk factor for death independent of age, sex and APACHE score, but not of SAPS and SOFA scores. Organ support techniques were used in the vast majority of patients and these were associated with poor prognosis. These results confirm the usefulness of the prognostic scales used on admission to the ICU but point to the need for individualized assessment of the expected benefit of ICU treatment in patients aged ≥80 years, taking into account not only age but also a range of other factors, including comorbidities, previous treatment and frailty.

## Figures and Tables

**Table 1:** Demographic and clinical characteristics of patients aged ≥80 years treated in the ICU with comparison across age groups and according to the outcome of treatment (death or survival).

Variables	All	Age Groups				Treatment Outcome		
		80–84	85–89	90+	*p*	Death	Survival	*p*
Demographics								
Female gender, *n*/N (%)	259/476 (54.4%)	126/249 (50.6%)	64/120 (53.3%)	69/107 (64.5%)	0.053	192/364 (52.6%)	67/112 (59.8%)	0.181
Age, median (min–max)/N	84 (80–103)/476	82 (80–84)/249	86 (85–89)/120	92 (90–103)/107	<0.001	85 (80–103)/364	83 (80–98)/112	0.107
ICU admission								
PTAiIT priority of ICU admission, median (min–max)/N	3 (1–4)/324	3 (1–4)/181	3 (1–4)/68	3 (1–4)/75	0.157	2 (1–4)/241	3 (1–4)/82	0,051
Surgery, *n*/N (%)	140/476 (25.8%)	68/249 (27.3%)	26/120 (21.7%)	29/107 (27.1%)	0.482	107/346 (30.9%)	33/112 (29.5%)	0.324
ED, *n*/N (%)	160/476 (33.6%)	69/249 (27.7%)	36/120 (30.0%)	55/107 (51.4%)	<0.001	114/363 (31.4%)	45/112 (40.2%)	0.085
COVID-19, *n*/N (%)	82/476 (17.2%)	48/249 (19.3%)	22/120 (18.3%)	12/107 (11.2%)	0.170	62/363 (17.1%)	20/112 (17.9%)	0.849
Co-morbidities and assessment of prognosis								
Ischemic heart disease, *n*/N (%)	182/476 (38.2%)	108/249 (43.4%)	39/120 (32.5%)	35/107 (32.7%)	0.054	135/363 (37.2%)	47/112 (40.2%)	0.364
Myocardial infarction, *n*/N (%)	93/476 (19.5%)	52/249 (20.9%)	27/120 (22.5%)	14/107 (13.1%)	0.150	72/363 (19.8%)	21/112 (18.8%)	0.800
Diabetes, *n*/N (%)	159/476 (33.4%)	38/249 (35.7%)	40/120 (33.3%)	30/107 (28.0%)	0.368	121/363 (33.3%)	38/112 (33.9%)	0.907
Chronic kidney disease, *n*/N (%)	123/476 (25.8%)	58/249 (23.3%)	33/120 (27.5%)	32/107 (29.9%)	0.379	95/363 (25.9%)	28/112 (25%)	0.850
Chronic hemodialysis, *n*/N (%)	10/476 (2.1%)	8/249 (3.2%)	2/120 (1.7%)	0/107 (0.0%)	0.142	9/363 (2.5%)	1/112 (0.9%)	0.307
Cancer, *n*/N (%)	89/476 (18.7%)	49/249 (19.7%)	22/120 (18.3%)	18/107 (17.0%)	0.812	67/363 (18.5%)	22/112 (19.6%)	0.779
COPD, *n*/N (%)	48/476 (10.1%)	28/249 (11.2%)	12/120 (10.0%)	8/107 (7.5%)	0.556	41/363 (11.3%)	7/112 (6.3%)	0.121
Obesity, *n*/N (%)	90/280 (32.1%)	64/164 (39.0%)	16/54 (29.6%)	10/62 (16.1%)	0.004	68/218 (31.2%)	22/62 (35.5%)	0.523
Ischemic stroke, *n*/N (%)	60/476 (12.6%)	31/249 (12.4%)	17/120 (14.2%)	12/107 (11.2%)	0.795	46/363 (12.7%)	14/112 (12.5%)	0.962
Intracranial bleeding, *n*/N (%)	32/476 (6.7%)	15/249 (6.0%)	9/120 (7.5%)	8/107 (7.5%)	0.816	21/363 (5.8%)	11/112 (9.8%)	0.136
IHD, *n*/N (%)	22/476 (4.6%)	8/249 (3.2%)	6/120 (5.0%)	8/107 (7.5%)	0.208	16/363 (4.4%)	6/112 (5.4%)	0.676
Alcoholism, *n*/N (%)	6/476 (1.3%)	5/249 (2.0%)	1/120 (0.8%)	0	0.264	6/363 (1.7%)	0/112 (0.0%)	0.171
APACHE, median (min–max)/N	27 (8–53)/244	28 (8–53)/123	16 (9–47)/60	26 (10–46)/61	0.157	28 (11–53)/194	25 (8–44)/49	0.022
SAPS, median (min–max)/N	67.5 (8–107)/219	68 (16–107)/110	64 (28–107)/64	68 (8–105)/55	0.585	67 (8–107)/178	58 (24–104)/41	0.001
SOFA, median (min–max)/N	12 (1–101)/231	13 (1–101)/123	11 (2–19)/52	12 (2–18)/56	0.026	13 2-(101)/183	9 (1–101)/48	<0.001
CFS, median (min–max)/N	7 (2–9)/189	7 (2–9)/96	7 (2–9)/50	7 (2–9)/43	0.845	7 (2–9)/151	6 (2–8)/38	0.008
Treatment and events during ICU stay								
IMV, *n*/N (%)	432/476 (90.8%)	227/249 (91.2%)	112/120 (93.3%)	93/107 (86.9%)	0.237	344/363 (94.8%)	87/112 (77.7%)	<0.001
NIV, *n*/N (%)	9/476 (1.9%)	4/249 (1.6%)	3/120 (2.5%)	2/107 (1.9%)	0.840	8/363 (2.2%)	1/112 (0.9%)	0.374
HFNO, *n*/N (%)	38/476 (8.0%)	19/249 (7.6%)	10/120 (8.3%)	9/107 (8.4%)	0.957	24/363 (6.6%)	14/112 (12.5%)	0.045
Conventional oxygen therapy, *n*/N (%)	173/476 (36.3%)	86/249 (34.5%)	39/120 (32.5%)	48/107 (44.9%)	0.107	95/363 (26.2%)	78/112 (69.6%)	<0.001
Vasopressors or inotropes, *n*/N (%)	396/476 (83.2%)	226/249 (90.8%)	83/120 (69.2%)	87/107 (81.3%)	<0.001	313/363 (86.2%)	83/112 (74.1%)	0.003
CRRT, *n*/N (%)	67/476 (14.1%)	38/249 (15.3%)	23/120 (19.2)	6/107 (5.6%)	0.010	59/363 (16.3%)	8/112 (7.1%)	0.015
Detection of alert pathogen, *n*/N (%)	126/476(26.5%)	73/249 (29.3%)	28/120 (23.3%)	25/107 (23.4%)	0.337	99/363 (27.3%)	27/112 (24.1%)	0.507
Death, *n*/N (%)	363/475 (76.4%)	178/249 (71.5%)	103/120 (85.8%)	82/106 (77.4%)	0.009	-	-	-
Days of ICU stay *, median (min–max)/N	8 (0–382)/476	8 (0–382)/249	5 (0–86)/120	8 (0–63)/107	0.508	7 (0–90)/363	8 (0–382)/112	0.111

* Zero means a stay of less than 1 calendar day. N—number of people with available data, *n*—count, min—smallest value, max—largest value, ICU—intensive care unit, ED—hospital emergency department, COPD—chronic obstructive pulmonary disease, CFS—frailty scale, IMV—invasive mechanical ventilation, NIV—non-invasive mechanical ventilation, HFNO—high-flow intranasal oxygen therapy, CRRT—continuous renal replacement therapy.

**Table 2:** Multivariate logistic regression models for the risk of death.

Variables	Model 1 (N = 189, *p* = 0.012)		Model 2 (N = 103, *p* = 0.005)		Model 3 (N = 93, *p* = 0.013)		Model 4 (N = 92, *p* = 0.001)	
	OR (95%CI)	*p*	OR (95%CI)	*p*	OR (95%CI)	*p*	OR (95%CI)	*p*
Age	0.97 (0.89–1.05)	0.408	0.93 (0.82–1.06)	0.267	0.95 (0.82–1.10)	0.516	0.95 (0.83–1.09)	0.441
Female gender	0.51 (0.23–1.12)	0.089	0.43 (0.14–1.35)	0.143	0.44 (0.12–1.54)	0.192	0.73 (0.20–2.62)	0.627
CFS	1.34 (1.09–1.66)	0.005	1.51 (1.06–2.15)	0.022	1.45 (0.97–2.19)	0.066	1.35 (0.92–1.99)	0.115
APACHE	-	-	1.08 (1.01–1.15)	0.016	-	-	-	-
SAPS	-	-	-	-	1.04 (1.01–1.07)	0.010	-	-
SOFA	-	-	-	-	-	-	1.25 (1.08–1.44)	0.002

Odds ratios (ORs) and 95% confidence intervals (95%CIs) are shown for female sex (relative to male sex), a 1-year increase in age, and a 1-point increase in scores on the CFS, APACHE, SAPS and SOFA scales. N denotes the number of subjects with available data.

## Data Availability

All relevant data are included in the published article. No external datasets were created or used.

## Abbreviations

The following abbreviations are used in this manuscript:

PIM MSWiA	National Medical Institute of the Ministry of the Interior and Administration
ICU	Intensive Care Unit
CFS	Clinical Frailty Scale
APACHE	Acute Physiology and Chronic Health Calculation
SAPS	Simplified Acute Physiology Score
SOFA	Sequential Organ Failure Assessment
CRRT	Continuous Renal Replacement Therapy
IMV	Invasive Mechanical Ventilation
NIV	Non-Invasive Ventilation
HFNO	High-Flow Nasal Oxygen Therapy
ED	Emergency Department
COPD	Chronic Obstructive Pulmonary Disease
PTAiT	Polish Society of Anesthesiology and Intensive Therapy
IHD	Ischemic Heart Disease
PMID	PubMed Identifier
OR	Odds Ratio
CI	Confidence Interval
BMI	Body Mass Index
ECMO	Extracorporeal Membrane Oxygenation
VIP1	Study name on patients aged 80+ in ICU
VIP2	Study name on patients aged 80+ in ICU
